# Research on the Adhesion Performance of Fast-Melting SBS-Modified Emulsified Asphalt–Aggregate Based on the Surface Free Energy Theory

**DOI:** 10.3390/ma18153523

**Published:** 2025-07-27

**Authors:** Hao Zhang, Haowei Li, Fei Guo, Shige Wang, Jinchao Yue

**Affiliations:** 1 School of Architecture and Civil Engineering, Xinyang Normal University, Xinyang 464000, China; 2 School of Software, Henan Normal University, Xinxiang 453000, China; 3 School of Water Conservancy and Transportation, Zhengzhou University, Zhengzhou 450000, China

**Keywords:** fast-melting SBS, modified emulsified asphalt, surface free energy, adhesion performance

## Abstract

Aiming at the problems of complex process flow, high energy consumption, and difficult emulsification in the preparation of traditional SBS-modified emulsified asphalt, a preparation method of fast-melting SBS (referred to as SBS-T) modified emulsified asphalt based on the integration of modification and emulsification is proposed. Based on surface free energy theory, the contact angles between three rapid-melting SBS-modified emulsified asphalts with different dosages and three probe liquids (deionized water, glycerol, and formamide) were measured using the sessile drop method. The adhesion performance of the asphalt–aggregate system was studied by means of micromechanical methods. The evaluation indicators such as the cohesion work of the emulsified asphalt, the adhesion work of asphalt–aggregate, the spalling work, and the energy ratio were analyzed. The results show that the SBS-T modifier can significantly improve the thermodynamic properties of emulsified asphalt. With increasing modifier content, the SBS-T-modified emulsified asphalt demonstrated enhanced cohesive work, improved asphalt–aggregate adhesive work, and increased energy ratio, while showing reduced stripping work. At equivalent dosage levels, the SBS-T-modified emulsified asphalt demonstrates a slight improvement in adhesion performance to aggregates compared to conventional SBS-modified emulsified asphalt. The SBS-T emulsified modified asphalt provides an effective technical solution for the preventive maintenance of asphalt pavements.

## 1. Introduction

As an innovative material offering advantages such as low-temperature construction, energy efficiency, and environmental friendliness, emulsified asphalt is emerging as a key material for road preventive maintenance. By dispersing asphalt particles in aqueous phase to form an emulsion, it enables low-temperature pavement operations while effectively avoiding the high energy consumption and environmental pollution associated with conventional hot-mix asphalt [1,2,3]. However, conventional emulsified asphalt still exhibits significant deficiencies in core performance aspects, including high-temperature deformation resistance, low-temperature crack resistance, and aggregate interface bonding [4,5,6,7]. Particularly under heavy traffic loads and harsh climatic conditions, its service life and aging resistance deteriorate markedly, severely limiting large-scale applications in road maintenance projects [8,9,10]. Consequently, performance enhancement of emulsified asphalt has become a critical challenge requiring urgent resolution in pavement engineering materials. Emulsified asphalt primarily consists of asphalt, emulsifiers, and water, with emulsifiers playing a pivotal role in stabilizing the emulsion and preventing asphalt particle agglomeration [11,12,13]. Current preparation technologies mainly include conventional methods and high-speed shearing techniques, where the latter proves more suitable for industrial-scale production due to its higher efficiency. Research indicates that optimizing parameters such as emulsifier type, dosage, and asphalt shearing rate can significantly improve emulsified asphalt performance to meet diverse engineering requirements [14,15]. Regarding performance evaluation, storage stability, rheological properties, and aggregate adhesion constitute the key indicators determining its engineering applicability. Notably, the storage stability of emulsified asphalt directly affects its performance during transportation and storage, with insufficient stability leading to issues like stratification or demulsification [16,17,18]. Therefore, developing high-efficiency emulsifiers and refining emulsification processes have become focal research directions, which hold significant implications for promoting wider applications of emulsified asphalt in road engineering.

The addition of SBS modifiers into emulsified asphalt systems has led to significant improvements in pavement performance, particularly in enhancing rutting resistance and fatigue resistance, making SBS-modified emulsified asphalt an increasingly important research focus in road maintenance materials. Currently, the predominant production process adopts an “emulsification first, modification second” approach, where emulsified asphalt is first prepared before SBS modifiers are uniformly dispersed through mechanical stirring or high-speed shearing. However, this conventional method presents several practical challenges: the process complexity requiring additional high-speed shearing equipment results in high energy consumption and costs; the poor dispersibility of SBS modifiers due to their large particle size and complex molecular structure limits swelling and adsorption in emulsified asphalt, leading to suboptimal modification effects; and the relatively low storage stability often causes phase separation and sedimentation during long-term storage, adversely affecting construction quality. These limitations have hindered the widespread application of SBS-modified emulsified asphalt.

To address these fundamental issues, researchers have turned to SBS-T modifiers [19,20,21,22], whose rapid-melting modification technology offers key advantages as follows: significantly reduced melting time and accelerated swelling rate in asphalt achieve an optimal balance between melt index and modification effectiveness [23,24,25,26]. The modification mechanism involves base asphalt components thoroughly swelling SBS-T polymer ions during mixing, while the SBS-T polymer ions demonstrate excellent adsorption of base asphalt components. Under mechanical mixing, their swelling–adsorption interaction forms a uniform mixture of asphalt and SBS-T that rapidly achieves its final modification effect when subjected to physical forces from emulsification equipment. This innovative approach overcomes the limitations of conventional SBS modification while maintaining all the performance advantages of modified emulsified asphalt, presenting a promising solution for advanced pavement materials.

The adhesion performance of emulsified asphalt and aggregates is an important factor in ensuring the water stability and long-term performance of emulsified asphalt mixtures, which directly affects the service life and stability of the pavement. With the continuous development of emulsified asphalt technology and its wide application in road construction and maintenance, the issue of emulsified asphalt–aggregate adhesion has attracted increasing attention from researchers. The adhesion between emulsified asphalt and aggregates is not only closely related to the physical and chemical properties of asphalt and aggregates but also influenced by various factors such as environmental factors and construction techniques. Therefore, in-depth research on the mechanism of emulsified asphalt–aggregate adhesion performance, revealing its influencing factors, and proposing reasonable improvement measures are of great theoretical significance and practical application value for enhancing the water damage resistance and long-term stability of asphalt pavement. In the research on the adhesion performance of emulsified asphalt and aggregates, many scholars have proposed different theories to explain this process. Mechanical theory holds that the adhesion effect stems from the pore interlocking and mechanical interlocking between asphalt and aggregates [27]. The chemical bond theory points out that a chemical reaction may occur between asphalt and aggregates to generate new substances, thereby enhancing the adhesion effect [28]. The molecular orientation theory holds that the asphalt active molecules are arranged in a specific direction within the mixed system and attract the polar components on the aggregate surface, thereby generating adhesion forces [29]. The electrostatic theory holds that aggregates and asphalt carry charges of opposite attraction and exhibit adhesion properties under the action of Coulomb force. The surface free energy theory emphasizes the variation in free energy between asphalt and aggregates and holds that reducing the free energy at the interface between the two is conducive to the improvement of adhesion [30].

Previous studies have mostly focused on hot-mix-modified asphalt, which requires heating to a high temperature of 160–180 °C for modification and construction. Its modification processes, such as the swelling and cross-linking of SBS with asphalt, rely on a high-temperature environment. Moreover, the adhesion with aggregates occurs in a high-temperature molten state, and the adhesion interface is significantly affected by factors like temperature decay and oxidative aging. The SBS-T-modified emulsified asphalt in this study is prepared through an “integrated modification-emulsification process”; under normal temperature conditions, the SBS-T modifier and asphalt directly form an oil-in-water emulsion through the synergistic effect of emulsifiers. Its interface interaction mechanism involves a dynamic process including emulsion demulsification, water evaporation, and asphalt film formation, which is fundamentally different from the high-temperature molten adhesion of hot-mix asphalt. This article investigates the adhesion performance between SBS-T-modified emulsified asphalt and aggregates (limestone and granite). Based on surface free energy theory, thermodynamic parameters such as work of cohesion, work of adhesion, work of debonding, and energy ratio were calculated using the sessile drop method and surface energy theory. The results provide an effective technical solution for preventive maintenance of asphalt pavements, along with theoretical support for the broader application of SBS-T-modified emulsified asphalt in road engineering.

## 2. Theoretical Background

The surface free energy theory explains the mechanisms of asphalt–aggregate adhesion and debonding from an interfacial energy perspective. This theory defines the energy required to separate materials or form new interfaces as surface energy and quantitatively describes the adhesion-debonding process by analyzing the interfacial energy changes among the three phases: asphalt, aggregate, and water. Based on parameters such as work of adhesion and work of debonding, water stability evaluation indices can be established, providing theoretical support for studying moisture damage mechanisms and developing anti-stripping materials.

The Generalized van Oss-Chaudhury (GvOC) model is the most widely used surface energy model in the field of road asphalt materials. The test object of this study is the asphalt film formed after demulsification of SBS-T-modified emulsified asphalt. Its surface state is affected by the demulsification process of the emulsion, and residual emulsifier molecules may be present, resulting in a heterogeneous polar interface. The contact angle measurement of the GvOC model is based on the interaction between probe liquids and the asphalt film at room temperature, which is more consistent with the room-temperature and water-based application scenarios of emulsified asphalt. According to GvOC theory, the surface free energy of a solid material can be divided into polar and nonpolar components, with the polar component further classified into Lewis acid and Lewis base fractions, as shown in Equation (1).

(1) $$\gamma^{total}=\gamma^{LW}+\gamma^{AB}=\gamma^{LW}+2\sqrt{\gamma^{+}\gamma^{-}}$$

where $\gamma^{total}$ is the total surface energy of the material. $\gamma^{LW}$ is the nonpolar dispersive component of the surface energy. $\gamma^{AB}$ is the polar component (acid–base component). $\gamma^{+}\;$ is the Lewis acidic component. $\gamma^{-}$ is the Lewis basic component.

The National Cooperative Highway Research Program (NCHRP RRD 316) summarized the main test methods for asphalt surface energy, including Atomic Force Microscopy (AFM), Nuclear Magnetic Resonance (NMR), Inverse Gas Chromatography (IGC), the Wilhelmy plate method, and the sessile drop method. Among these, the sessile drop method has been widely adopted due to its low testing requirements, as it can effectively characterize the wettability and surface energy of solid asphalt. By examining the relationship between the contact angle of the test liquid and the surface energy of solid asphalt, an expression consistent with Young’s equation can be derived, as shown in Equation (2).

(2) $$\gamma_{s}=\gamma_{sl}+\gamma_{l}\cos\theta$$

where $\gamma_{s}$ is the solid surface energy.

$\gamma_{l}$ is the surface energy of the liquid. $\gamma_{sl}$ is the interfacial free energy between solid and liquid.

According to thermodynamic theory, the work required to divide a homogeneous asphalt material into two new interfaces is called the cohesive work of asphalt. Its value is equal to twice the surface energy, and the specific calculation formula is shown in (3):

(3) $$W_{BB}=2\gamma_{B}$$

where *B* is aggregate.

The work of adhesion refers to the energy released when water displaces the asphalt–aggregate interface, and its magnitude depends on the free energy of the asphalt–aggregate interface, the asphalt–water interface, and the aggregate–water interface. The calculation formula is shown in Equation (4). The greater the absolute value of the work of adhesion, the more easily water can displace the asphalt at the asphalt–aggregate interface, thereby reducing the resistance to moisture damage.

(4) $$W_{AB}=2\sqrt{\gamma_{A}^{LW}\gamma_{B}^{LW}}+2\sqrt{\gamma_{A}^{+}\gamma_{B}^{-}}+2\sqrt{\gamma_{A}^{-}\gamma_{B}^{+}}$$

where *A* is asphalt and *B* is aggregate.

The stripping work refers to the energy released when water displaces the asphalt–aggregate interface. Its magnitude depends on the free energy of the asphalt–aggregate interface, the asphalt–water interface, and the aggregate–water interface, as calculated in Equation (5). The larger the absolute value of the stripping work, the more easily water can displace the asphalt at the asphalt–aggregate interface, thereby reducing the resistance to moisture damage.

(5) $$\begin{array}{ll} W_{aBWA}^{wet} & =2\gamma_{W}^{LW}+2\sqrt{\gamma_{B}^{LW}\gamma_{W}^{LW}}-2\sqrt{\gamma_{A}^{LW}\gamma_{W}^{LW}}\\ & +4\sqrt{\gamma_{W}^{+}\gamma_{W}^{-}}-2\sqrt{\gamma_{W}^{+}}\left(\sqrt{\gamma_{B}^{-}}+\sqrt{\gamma_{A}^{-}}\right)\\ & -2\sqrt{\gamma_{W}^{-}}\left(\sqrt{\gamma_{B}^{+}}+\sqrt{\gamma_{A}^{-}}\right)+2\sqrt{\gamma_{B}^{+}\gamma_{A}^{-}}\\ & +2\sqrt{\gamma_{B}^{-}\gamma_{A}^{+}} \end{array}$$

where *w* is water.

The work of adhesion and the work of debonding only evaluate the adhesive performance between asphalt and aggregate under dry and wet conditions from a single aspect, failing to comprehensively reflect the moisture damage resistance of asphalt mixtures. Therefore, Bhasin et al. [31] proposed the energy ratio parameter based on extensive experimental research, which is defined as the ratio of the work of adhesion under dry conditions to the work of debonding in the presence of water. This parameter is used to assess the moisture susceptibility of asphalt mixtures—the higher the energy ratio value, the stronger the resistance to moisture damage.

(6) $$ER=\frac{W_{AB}}{W_{aBWA}^{wet}}$$

## 3. Materials and Methodologies

### 3.1. Preparation of SBS-T-Modified Emulsified Asphalt

#### 3.1.1. Matrix Asphalt

The type and quality of asphalt will directly affect the performance of emulsified asphalt. Due to differences in chemical composition (such as the ratio of aromatics to saturates) and intrinsic polarity, base asphalts of different grades or from different sources will have a certain impact on the improvement effect of SBS-T modifier on adhesion performance; low-grade asphalts, with low aromatic content and high viscosity, allow SBS-T to more easily form hydrogen bonds with asphaltenes after dispersion, leading to a more significant improvement in adhesion to alkaline aggregates, but the improvement for acidic aggregates may be limited. High-grade asphalts, with high aromatic content and low viscosity, facilitate the rapid swelling of SBS-T and the exposure of polar groups, resulting in a more obvious increase in interfacial bonding energy, and the dosage needs to be adjusted to balance performance. In this paper, 70# base asphalt was selected. Its basic indicators were determined according to the requirements of “Test Procedures for Asphalt and Asphalt Mixtures in Highway Engineering” (JTG E20-2011) [32]. The test technical indicators are shown in Table 1, and all the test results meet the requirements.

#### 3.1.2. Asphalt Modifier

This study utilizes SBS-T modifier and SBS modifier produced by Guolu Gaoke Engineering Technology Institute Co., Ltd. (No. 30 College Rd., Haidian District, Beijing, China). The differences in physical properties between SBS-T and SBS primarily manifest in two key indicators: melt index and particle size.

Melt index and particle size are crucial physical parameters for modifiers. A higher melt index indicates better processing performance, while smaller particle sizes enhance the swelling and adsorption interactions between the components. This results in more thorough modification of the base asphalt and improved stability of the resulting modified emulsified asphalt.

**Figure 1:**
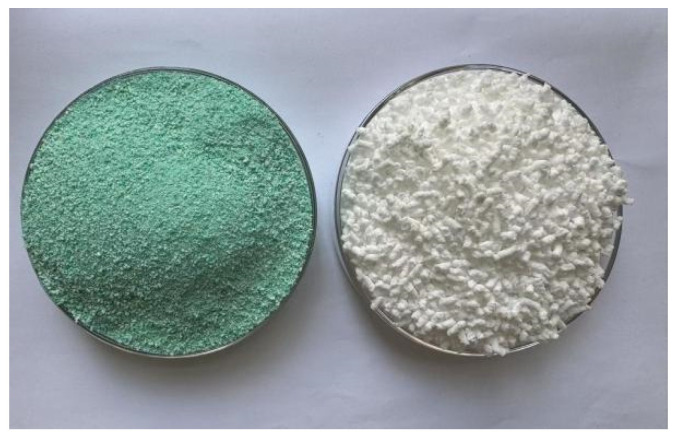
SBS-T (**left**) and SBS (**right**) modifier.

The SBS-T modifier used in this article has a particle size of 0.2 mm and a melt index of 1.5–2.5 g/10 min, whereas the SBS modifier has a particle size of 0.5–3 mm and a melt index of 0.82 g/10 min. Pictures of the two modifiers are shown in Figure 1.

#### 3.1.3. Modified Emulsified Asphalt Preparation

The preparation process of emulsified asphalt can traditionally be divided into two main technical routes: the process of modification first and then emulsification and the process of emulsification first and then modification. Both of these two processes are applied in the preparation of SBS-modified emulsified asphalt but each has its advantages and disadvantages.

**Figure 2:**
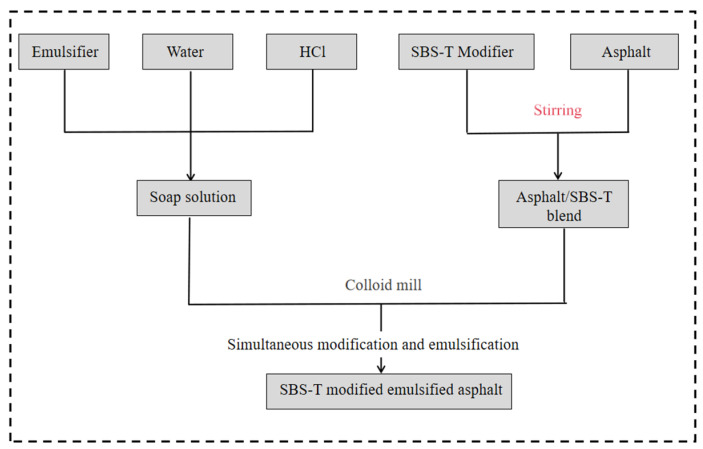
Integrated preparation process of modification and emulsification.

With the increasing demand for the performance of SBS-modified emulsified asphalt, the deficiencies of traditional processes in improving efficiency, reducing energy consumption, and enhancing product performance have gradually emerged. In response to these deficiencies, this paper organically combines the modification and emulsification processes. Through the integrated process of modification and emulsification, the aim is to reduce energy consumption and shorten the production cycle. The specific preparation process is shown in Figure 2.

This research adopts an integrated modification and emulsification process to prepare SBS-T-modified emulsified asphalt with 4%, 5%, and 6% dosage as the experimental groups, while conventional SBS-modified emulsified asphalt with 4%, 5%, and 6% dosage serves as the control groups. The preparation method for SBS-T-modified emulsified asphalt is as follows:

First, pretreatment is required. The pre-dispersed SBS-T modifier is added to the base asphalt. At this stage, the SBS-T modifier is thoroughly stirred at a temperature of 150–190 °C to ensure sufficient swelling and effective interaction with the components of the base asphalt. This process guarantees uniform dispersion of the SBS-T polymer in the asphalt, thereby improving the homogeneity and stability of the modified asphalt. The principle of stirring modification is that the SBS-T modifier, being a polymeric ion, undergoes full swelling when mixed with the base asphalt. Simultaneously, the SBS-T polymeric ions exhibit strong adsorption with the base asphalt components. Under mechanical stirring, the swelling and adsorption interactions between the two result in a mixture of asphalt and SBS-T. When subjected to the physical shear forces of the emulsification machine, the final modification effect is rapidly achieved.

Subsequently, the asphalt–SBS-T mixture is added to the emulsification test machine along with the emulsifier. The same cationic emulsifier is used, diluted with water at a ratio of 1:10. Diluted hydrochloric acid is gradually added to adjust the pH of the emulsifier solution to 2–2.5 using pH test paper. At this stage, high-speed shearing simultaneously accomplishes both emulsification and modification. The shear speed of the emulsification test machine is controlled at 5000 rpm, with the discharge temperature maintained at 60–70 °C. After emulsification, the mixture is cooled to 20–25 °C using a cooling device and stored in a sealed container to prevent water evaporation or emulsifier degradation.

When the temperature exceeds 190 °C, although it can reduce the viscosity of asphalt, it will significantly accelerate the volatilization of light components in asphalt (such as saturates and aromatics), causing the asphalt to harden and its low-temperature performance to deteriorate. The discharge temperature of emulsified asphalt needs to match the normal-temperature operations in subsequent construction (such as slurry seal). A discharge temperature of 60–70 °C allows the emulsion to naturally cool to room temperature (around 25 °C) during storage, avoiding uneven water evaporation caused by excessive temperature differences and ensuring the leveling property during construction.

Throughout the preparation process, temperature control is critical to ensure the effectiveness of the emulsifier and the uniformity of the modified asphalt. Additionally, the operational conditions of the stirring and shearing equipment must be optimal to guarantee product dispersity and stability. Finally, the finished product is tested for storage stability, particle size distribution, and various performance indicators to ensure the emulsified asphalt meets design requirements, providing a technical basis for subsequent mass production. The storage stability of SBS-T-modified emulsified asphalt was tested with reference to Method T 0655 in Specifications for Bitumen and Bituminous Mixtures in Highway Engineering (JTG-E20-2011): The emulsion was placed in a 500 mL measuring cylinder and left to stand at (25 ± 1) °C for 5 days. Then, the emulsion samples from the upper 1/3 and lower 1/3 of the measuring cylinder were taken to test the content of evaporation residues and the stratification rate was calculated. The results showed that the stratification rate of samples prepared by the integrated process was 2.1–2.5% (n = 3 parallel experiments), while that of the control samples prepared by the traditional “modification first, then emulsification” process was 4.8–5.3%.

### 3.2. Contact Angle Measurement Methods

#### 3.2.1. Test Method

**Figure 3:**
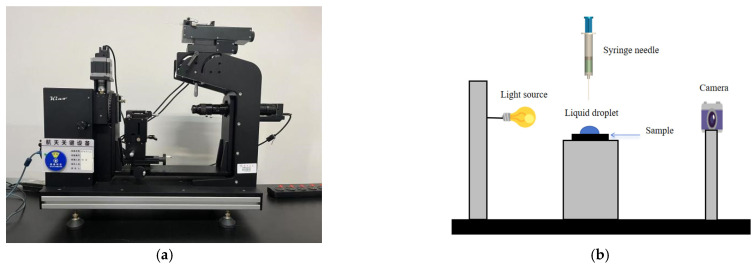
Optical contact angle measuring instrument: (**a**) physical diagram; (**b**) schematic diagram (right).

In this experiment, the SL200KS optical contact angle and interfacial tension measuring instrument was used to measure the contact angle of the sample. This instrument consists of a light source system, a video acquisition system, and an analysis system. The main principle is to capture the contact process between the test liquid and the sample through a high-resolution camera and then lock the image as a static image through the contact angle analysis software. The static contact angle was obtained by using the circular fitting method. The physical test diagram and principle are shown in Figure 3.

The contact angle of the samples was measured by the lying drop method, and distilled water, glycerol, and formamide were selected as the test liquids. To prevent interference between liquids, three samples of each type of asphalt were prepared, and one sample of each liquid was used. To ensure the accuracy of the test results, each sample was measured five times at different positions, and the average value was taken to reduce the variation level of the test results. The surface free energy of the tested liquid and its components are shown in Table 2.

This article investigates the adhesion characteristics of limestone and granite as aggregate samples with emulsified asphalt. The static drop method was employed to measure the contact angles of distilled water, glycerol, and formamide on the surface of the aggregates. Based on surface energy theory, the surface energy and its components of the aggregates were calculated, with detailed results presented in Table 3.

#### 3.2.2. Sample Preparation

Since emulsified asphalt exists in a liquid state containing water at ambient temperature, it is imperative to isolate the evaporation residue of SBS-T-modified emulsified asphalt for characterization. The standard methodology for obtaining the evaporation residue involves a controlled heating process, implemented as follows.

A precisely measured quantity of emulsified asphalt specimen is transferred into a pre-weighed evaporation crucible. The crucible is then positioned on a heating plate or within a forced-air oven maintained at a regulated temperature (163 ± 3 °C or 105 ± 5 °C, as specified by experimental protocols) within a fume hood. During heating, continuous agitation is applied to ensure homogeneous heat distribution and prevent localized thermal degradation. The process is sustained until complete moisture evaporation is achieved, as evidenced by the cessation of bubbling. Subsequently, the crucible is transferred to a desiccator for equilibration to ambient temperature, followed by gravimetric analysis to determine the percentage of residual mass relative to the initial specimen. This protocol ensures complete solvent removal and residue solidification, thereby providing an accurate quantification of the asphalt content within the emulsified matrix.

**Figure 4:**
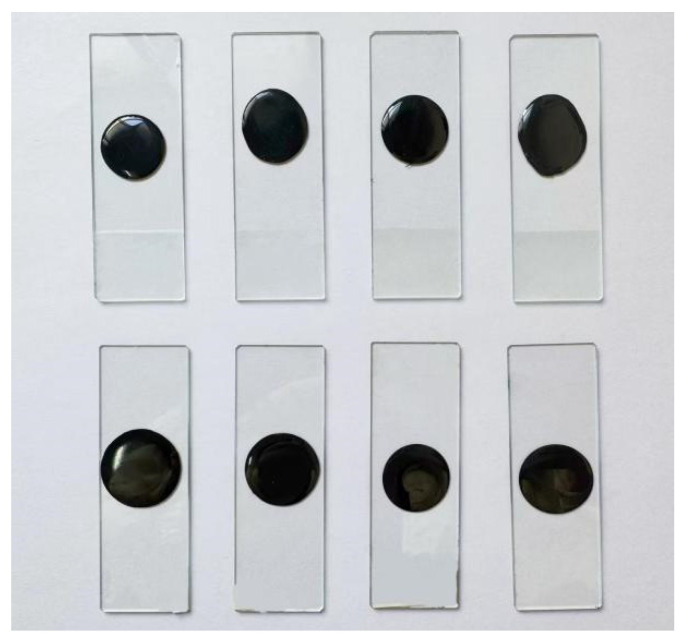
Contact angle samples of asphalt binders.

For contact angle measurements, the evaporation residue is thermally treated to achieve a fluid state. A glass rod is employed to transfer a droplet of the molten residue onto a microscope slide, which is then subjected to isothermal conditioning at 150 °C for 20 min in a convection oven to facilitate planarization through viscous flow. A temperature of 150 °C is a critical temperature range where asphalt is in a viscous flow state. At this temperature, the viscosity of asphalt decreases significantly. Under the combined action of gravity and surface tension, asphalt spontaneously fills tiny depressions and eliminates local protrusions on the glass slide through viscous flow, and the 20-min constant temperature duration ensures that the flow reaches a dynamic equilibrium. The specimen is subsequently cooled to 25 °C under ambient conditions and stored in a desiccator prior to analysis. The contact angle samples are illustrated in Figure 4.

## 4. Results and Analysis

### 4.1. Cohesive Work

**Figure 5:**
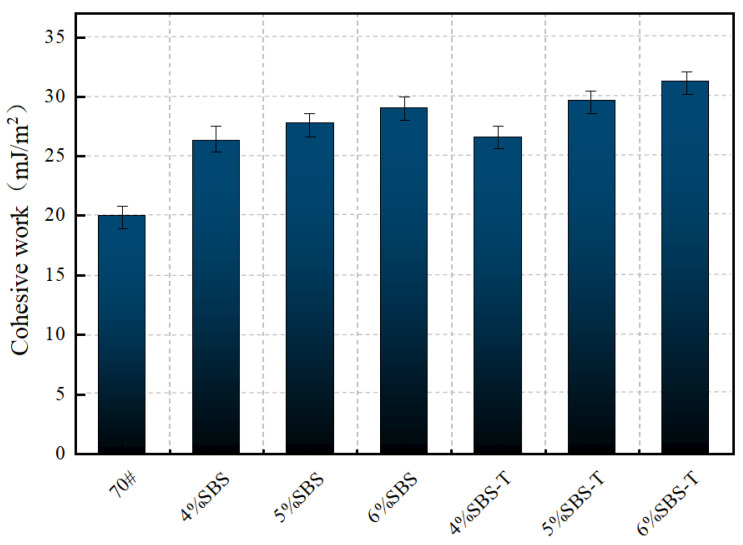
Cohesive work of SBS- and SBS-T-modified emulsified asphalt.

The cohesion work of asphalt serves as a key indicator of its cohesive performance. The magnitude of cohesion work is equivalent to twice the surface energy, with higher values corresponding to enhanced crack resistance. This implies improved resistance to water penetration through cracks at the asphalt–aggregate interface. The surface free energy of the tested asphalt and its components are shown in Table 4. Figure 5 presents the cohesion work of SBS- and SBS-T-modified emulsified asphalt.

**Figure 6:**
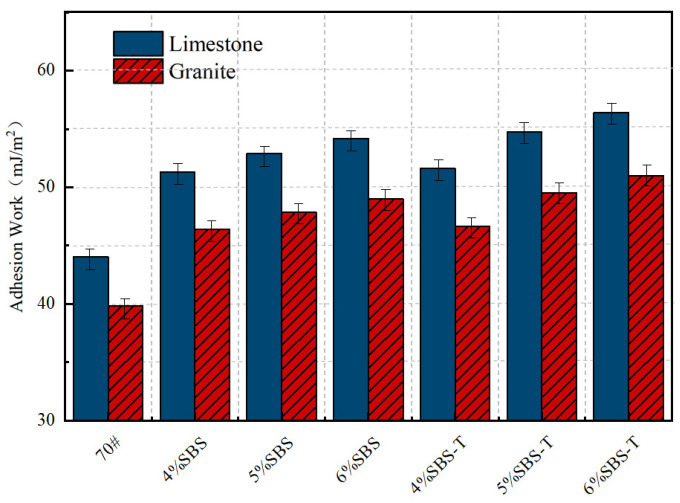
Adhesion work of SBS- and SBS-T-modified emulsified asphalt to aggregates.

As illustrated in Figure 6, the cohesion work exhibits a gradual increase with rising dosages of both SBS- and SBS-T-modified emulsified asphalt. The base emulsified asphalt demonstrates the lowest cohesion energy (20.04 mJ/m^2^). However, at a 6% dosage, the cohesion energy of SBS- and SBS-T-modified emulsified asphalt increases significantly to 29.02 mJ/m^2^ and 31.28 mJ/m^2^, respectively, indicating a substantial improvement in interfacial adhesion due to the modifier.

For SBS-modified emulsified asphalt, the cohesion energy rises progressively from 26.3 mJ/m^2^ (4% dosage) to 29.02 mJ/m^2^ (6% dosage). This trend suggests that increasing the SBS content elevates the surface free energy of the emulsified asphalt, particularly its dispersion component, thereby strengthening van der Waals forces between interfacial molecules and enhancing adhesion performance. However, the rate of increase in cohesion work moderates at higher dosages, likely attributable to the larger particle size of SBS, which restricts its swelling and adsorption efficiency within the base asphalt.

In contrast, SBS-T-modified emulsified asphalt shows a slight improvement in cohesive performance compared to SBS at the same dosage. At 6%, its cohesive work reaches 31.28 mJ/m^2^, which indicates good dispersibility and fluidity during the emulsification process, resulting in a uniform and stable emulsified asphalt system. The observed increase in cohesion work reflects elevated surface free energy, particularly in the polar component. The higher dosages of SBS and SBS-T facilitate greater exposure of polar functional groups (e.g., hydroxyl and carboxyl groups) on the asphalt surface, thereby improving adhesion to aggregate surfaces.

### 4.2. Adhesion Work

Adhesion work serves as a critical indicator for evaluating the adhesion performance between asphalt and aggregates in an anhydrous state. Its magnitude is intrinsically linked to the surface energy of the asphalt, the surface energy of the aggregates, and the interfacial energy between the two phases. A higher adhesion work value corresponds to greater stability in the asphalt–aggregate system and stronger interfacial adhesion. The comparative results are presented in Figure 6.

As illustrated in Figure 6, the adhesion energy of SBS-T-modified emulsified asphalt exhibits a notable increase as the modifier dosage rises from 4% to 6%, demonstrating both a higher improvement amplitude and a slight improvement final adhesion energy value. In comparison to conventional SBS, SBS-T not only achieves higher adhesion values at each dosage but also displays a more pronounced growth trend. This enhanced performance can be attributed to the finer particle size of SBS-T (200 μm), which substantially increases the interfacial contact area with the base asphalt, thereby amplifying swelling and adsorption effects. Such microstructural advantages contribute to the more significant improvement in adhesion work observed with SBS-T. Furthermore, the higher melt index of SBS-T (1.5–2.5 g/10 min) enhances its fluidity and dispersibility during emulsification, facilitating a more uniform distribution within the asphalt matrix under high-speed shear conditions. This homogeneous dispersion not only strengthens interfacial bonding but also optimizes the exposure of polar groups and dynamic adsorption capacity. Notably, at a higher dosage (6%), the adhesion work of SBS-T experiences a substantial increase, suggesting more comprehensive molecular coverage and chemical bonding at the aggregate interface. These findings indicate that SBS-T is particularly suitable for high-dosage applications and exhibits greater potential in enhancing the interfacial performance of emulsified asphalt.

The enhancement of adhesion work directly correlates with improved interfacial bonding between emulsified asphalt and aggregates, which is crucial for key macroscopic properties such as moisture resistance, anti-stripping performance, and long-term durability. In contrast to conventional SBS, SBS-T-modified emulsified asphalt demonstrates a slight improvement in resistance to water-induced damage and fatigue.

According to the research findings of Grenfell et al. [33], the bonding strength between modified asphalt and limestone aggregate is a slight improvement to that with granite, primarily attributed to differences in aggregate surface energy. Limestone, due to its higher surface energy characteristics, exhibits better asphalt adhesion performance, a conclusion consistent with the findings of this study. From a chemical composition perspective, limestone is an alkaline aggregate rich in calcite, while granite is acidic with silica as its main component. Given that asphalt itself exhibits weakly acidic properties, its bonding with limestone is primarily achieved through chemical adsorption, whereas its adhesion to granite relies on physical adsorption. This characteristic endows the asphalt–limestone system with relatively stable adhesion performance.

### 4.3. Stripping Work

**Figure 7:**
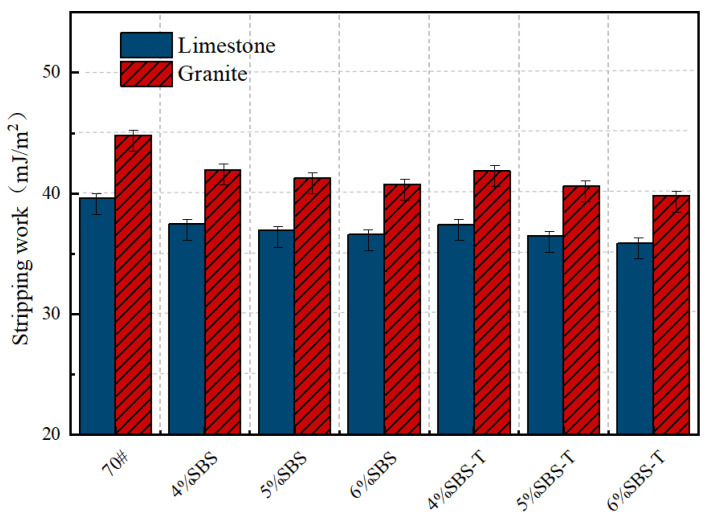
Stripping work of SBS- and SBS-T-modified emulsified asphalt to aggregates.

Moisture is a primary factor contributing to adhesion failure in asphalt–aggregate systems. When water infiltrates the asphalt mixture through cracks, aggregates exhibit a stronger affinity for highly polar water molecules, leading to the detachment of asphalt from the aggregate surface. The energy released during this process is defined as the spalling work. Figure 7 presents the spalling work for different modified emulsified asphalts in combination with two aggregate types—limestone and granite.

As illustrated in Figure 7, the spalling work of SBS-modified emulsified asphalt decreases progressively with increasing modifier dosage. Notably, the absolute spalling work values for SBS-T are consistently lower than those of conventional SBS across all dosages, indicating it has slightly stronger moisture resistance. This enhancement can be attributed to the SBS-T modifier’s ability to promote denser molecular bonding at the asphalt–aggregate interface, forming a more stable interfacial structure. Furthermore, as the dosage increases, SBS-T effectively mitigates water infiltration by elevating the free energy at the asphalt–water interface, thereby reducing spalling work and improving moisture resistance.

The high dispersibility of SBS-T ensures a more uniform interfacial distribution, minimizing weak interfacial zones susceptible to moisture intrusion. Additionally, its elevated polar component enhances chemical adsorption between the asphalt and polar functional groups on the aggregate surface. This adsorption mechanism partially counteracts the disruptive effects of water on interfacial adhesion, contributing to reduced spalling work.

Both SBS- and SBS-T-modified asphalts exhibit a decreasing trend in absolute spalling work with increasing dosage, suggesting that higher modifier content enhances moisture damage resistance. However, SBS-T consistently outperforms SBS, demonstrating lower spalling work values at all dosages. Mechanistically, the reduction in spalling work reflects increased resistance to asphalt displacement by water, which is closely associated with modifier-induced optimization of asphalt surface free energy.

The excellent performance of SBS-T stems from its enhanced dispersibility and swelling adsorption capacity, which slightly increases the free energy at the asphalt–water interface, thereby reducing water-induced interfacial damage. In contrast, conventional SBS exhibits limitations in interfacial binding strength and molecular dynamic rearrangement, resulting in only marginal improvements in moisture resistance at higher dosages.

### 4.4. Energy Ratio

**Figure 8:**
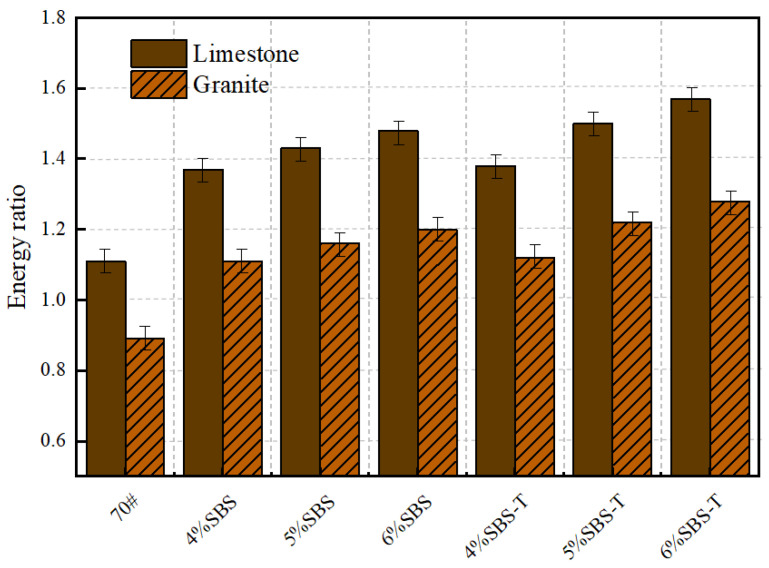
Energy ratio of SBS- and SBS-T-modified emulsified asphalt to aggregates.

The energy ratio is defined as the ratio between the adhesion work of asphalt in a dry state and the stripping work of the asphalt–aggregate–water system in a hydrated environment. To enhance the water stability of asphalt mixtures, it is essential to maximize the adhesion work between asphalt and aggregate while minimizing the spalling work. A comparative analysis of the interfacial energy characteristics for different asphalt–aggregate combinations is illustrated in Figure 8.

As depicted in Figure 8, the energy ratio of SBS-T-modified emulsified asphalt consistently exceeds that of SBS-modified asphalt across all dosage levels. This trend suggests that SBS-T modification significantly improves the resistance of emulsified asphalt to water-induced damage. As the content of the modifier increases, the energy ratios of both SBS and SBS-T increase, but SBS-T maintains slightly stronger performance at all addition levels, indicating that its effect in reducing moisture sensitivity is enhanced.

The observed increase in energy ratio can be attributed to the improved molecular bonding stability at the asphalt–aggregate interface due to modifier incorporation. The SBS-T modifier, characterized by its fine particle size, high dispersion efficiency, and robust dynamic adsorption capacity, facilitates a more uniform interfacial distribution. Consequently, under moisture intrusion, the likelihood of molecular structure disruption is reduced, leading to lower energy dissipation and an overall elevation in the energy ratio.

## 5. Conclusions

This study investigates the bonding performance between SBS-T-modified emulsified asphalt, prepared via an integrated modification and emulsification approach, and limestone as well as granite aggregates, based on micromechanical methods. The contact angle between asphalt and aggregates was measured using the sessile drop method. Thermodynamic parameters between the emulsified asphalt and aggregates were calculated based on the surface free energy theory. The main conclusions are as follows:

(1) Compared to traditional SBS modifiers, the SBS-T modifier exhibits smaller particle size and higher melt index, enabling more sufficient swelling and adsorption in asphalt, thereby improving the dispersibility and uniformity of emulsified asphalt more effectively. The selection of modifiers plays a crucial role in optimizing the performance of emulsified asphalt. The integrated modification and emulsification process combines modification and emulsification steps organically, not only reducing process steps and preparation energy consumption but also enhancing the storage stability of emulsified asphalt.

(2) With the increase in modifier content, the cohesive work and adhesive work of the two modified emulsified asphalts significantly improve, while the stripping work gradually decreases. Among them, SBS-T-modified asphalt demonstrates a slight improvement in performance at the same dosage. A clear dose–effect relationship exists between modifier content and performance indicators, with SBS-T-modified asphalt showing greater performance improvements within the 4–6% dosage range, indicating better modification effects.

(3) By comparing indicators such as bonding work, stripping work, and energy ratio between asphalt and aggregates, the bonding performance of different types of asphalt with aggregates follows the order: SBS-T-modified emulsified asphalt > SBS-modified emulsified asphalt > 70# base asphalt. For the same asphalt with different aggregates, the bonding performance follows the order: limestone > granite. Among the various combinations, the SBS-T-modified emulsified asphalt–limestone aggregate system exhibits the best resistance to moisture-induced damage.

## Figures and Tables

**Table 1:** Basic technical specifications for 70# asphalt.

Technical Indicators	Unit	Measured Value	Test Methods
Penetration (25 °C)	0.1mm	65.7	T0604-2011
Ductility (15 °C, 5 cm/min)	cm	>100	T0605-2011
Softening point	°C	53.4	T0606-2011

The test methods are in accordance with JTG E20-2011.

**Table 2:** Surface free energy of liquid and the components.

Aggregate Type	Unit	$\gamma_{l}$	$\gamma_{l}^{LW}$	$\gamma_{l}^{AB}$	$\gamma_{l}^{+}$	$\gamma_{l}^{-}$
Distilled water	mJ/m^2^	72.8	21.8	51.0	25.5	25.5
Formamide	mJ/m^2^	58.0	39.0	19.0	2.28	39.6
Glycerol	mJ/m^2^	64.0	34.0	30.0	3.92	57.4

**Table 3:** Surface free energy of aggregates and the components.

Aggregate Type	Unit	$\gamma_{l}$	$\gamma_{l}^{LW}$	$\gamma_{l}^{AB}$	$\gamma_{l}^{+}$	$\gamma_{l}^{-}$
limestone	mJ/m^2^	42.17	32.72	9.45	1.55	14.39
granite	mJ/m^2^	35.51	26.96	8.55	1.67	10.92

**Table 4:** Surface free energy of asphalt and the components.

Asphalt Type	Unit	$\gamma_{l}$	$\gamma_{l}^{LW}$	$\gamma_{l}^{AB}$
70#	mJ/m^2^	9.31	8.04	1.27
4%SBS	mJ/m^2^	11.11	9.18	1.93
5%SBS	mJ/m^2^	11.72	9.55	2.17
6%SBS	mJ/m^2^	12.35	9.92	2.44
4%SBS-T	mJ/m^2^	11.41	9.36	2.05
5%SBS-T	mJ/m^2^	12.03	9.73	2.30
6%SBS-T	mJ/m^2^	12.68	10.1	2.58

## Data Availability

The original contributions presented in the study are included in the article; further inquiries can be directed to the corresponding author.
